# Identification of a conserved GNAT-family lysine acetyltransferase in *Streptococcus gordonii* involved in biofilm formation and oral colonization

**DOI:** 10.1128/jb.00102-26

**Published:** 2026-07-27

**Authors:** Joseph O'Brien, Flavia M. Saavedra, Irene Choi, Kyle J. McCulloch, Maria Martell, Alexandra M. Peterson, Christopher Staley, Mark C. Herzberg, Bruno P. Lima

**Affiliations:** 1 Department of Emergency Medicine, Denver Health 47804, Denver, Colorado, USA; 2 Department of Inflammation and Immunity, Lerner Research Institute, Cleveland Clinic Foundation 583189, Cleveland, Ohio, USA; 3 Department of Diagnostic and Biological Sciences, University of Minnesota School of Dentistry 160911, Minneapolis, Minnesota, USA; 4 Department of Biology, University of Florida 166765, Gainesville, Florida, USA; 5 Division of Basic & Translational Research, Department of Surgery, University of Minnesota 311866, Minneapolis, Minnesota, USA; 6 Department of Endodontics, University of Florida College of Dentistry 164889, Gainesville, Florida, USA; 7 Department of Oral Biology, University of Florida College of Dentistry 164889, Gainesville, Florida, USA; Dartmouth College Geisel School of Medicine, Hanover, New Hampshire, USA

**Keywords:** oral streptococci, biofilms, acetylation

## Abstract

*Streptococcus gordonii* is a gram-positive oral bacterium capable of adhering to a variety of biotic and abiotic surfaces and forming biofilms. To characterize physiological changes associated with biofilm formation in *S. gordonii*, we investigated the roles of two putative GCN5-related N-acetyltransferases (GNATs), SGO_2030 and SGO_2031, in *in vitro* biofilm formation on saliva-coated surfaces using the laboratory strain DL1. Our results demonstrate that SGO_2031, but not SGO_2030, seems to contribute to biofilm formation by modulating the abundance of extracellular polysaccharides within the biofilm matrix. This defect in biofilm formation observed by the deletion of SGO_2031 resulted in a significant fitness disadvantage during colonization of the murine oral cavity compared to the wild-type parent strain. Consistent with the role of *S. gordonii* as an early colonizer of tooth surfaces that influences oral biofilm community structure, inoculation with either the wild-type or the SGO_2031 mutant strain led to distinct alterations in the murine oral microbiome composition. Deletion of SGO_2031 also resulted in changes in protein acetylation patterns, as assessed by Western immunoblot analysis, supporting the role of this enzyme as an acetyltransferase. Given that SGO_2031 is conserved and widely distributed among streptococci, we propose naming this enzyme Streptococcal Lysine Acetyltransferase A (SktA).

**IMPORTANCE:**

Protein acetylation is a common posttranslational modification conserved across all domains of life. In bacteria, protein acetylation is carried out by homologs of the GCN5-related N-acetyltransferase (GNAT) family. GNATs catalyze the transfer of an acetyl group from acetyl-CoA to the ε-amino group of lysine residues on proteins. This process changes the charge and length of lysine residues, resulting in changes to protein function. *Streptococcus gordonii* is predicted to encode 17 GNAT homologs. Here, we report that one of them, SGO_2031 (SktA), plays an important role in *S. gordonii* biofilms.

## INTRODUCTION

In their natural environment, bacteria are commonly found in organized, multicellular, and often multispecies communities known as biofilms (1). In the oral cavity, streptococci form complex communities with hundreds of other bacterial species and comprise the majority of bacteria in newly formed biofilms on the tooth surface (i.e., dental plaque) (2, 3). An array of surface adhesin proteins, which bind different components of the salivary pellicle (the thin layer of salivary glycoproteins that forms on oral surfaces), mediate the attachment of bacteria to the tooth and other oral surfaces (4–6). Surface adhesins expressed by oral streptococci and other early colonizers of the oral biofilms also mediate the interaction with other members of the oral microbiota, facilitating their incorporation into the developing community (7, 8).

We have shown that *Streptococcus gordonii*, an early colonizer of dental plaque, monitors and regulates the presentation of surface adhesins (9), likely playing an important role in dental plaque development. For example, *in vitro*, *S. gordonii* downregulates the expression of the antigen I/II family of cell wall-anchored surface adhesins (SspA and SspB) upon attachment to a saliva-coated surface (10). Similarly, *sspA* and *sspB* gene expression in the human oral cavity also appears to respond to environmental cues as levels of *sspA* and *sspB* were higher in *S. gordonii* cells obtained from saliva compared to dental plaque-associated cells (9).

The mucin glycoprotein MUC5B, a major component of saliva and the salivary pellicle, has been shown to affect *S. gordonii* physiology by altering the expression of as many as 63 genes, including the cell wall-anchored adhesins *sspB* and *mbpA* (11). Of particular interest, when *S. gordonii* cells formed a biofilm on MUC5B-coated surfaces, genes encoding two putative Gcn5-related N-acetyltransferases (GNAT), SGO_2030 and SGO_2031, were downregulated, indicating that protein acetylation is likely influenced by the bacterial lifestyle (i.e., biofilm versus planktonic).

Protein acetylation is a common posttranslational modification conserved among all domains of life (12). In bacteria, protein acetylation has been shown to occur both enzymatically and non-enzymatically (13, 14). Enzymatic protein acetylation in bacteria is carried out by the GNAT family of acetyltransferases, which transfer an acetyl group from acetyl-CoA to the ε-amino group of lysines. This process alters residue charge and side-chain length, potentially affecting the stability of proteins and the interactions between proteins and nucleic acids (15).

Since the first bacterial acetylome was reported in 2008 (16), mass spectrometry has been extensively used to identify acetylated proteins. We now know that multiple cellular processes contain proteins that can be acetylated, including central metabolism, transcription, and translation; therefore, protein acetylation broadly impacts bacterial physiology (17, 18). Among oral bacteria, protein acetylation has been shown to contribute to acid production and the formation of biofilms in *Streptococcus mutans* (19, 20) and gingipain biogenesis in *Porphyromonas gingivalis* (21, 22). Global acetylome data from these two organisms suggest that protein acetylation may also impact many other cellular processes.

Here, we report that a previously uncharacterized lysine acetyltransferase (SGO_2031) affects the presence of extracellular polysaccharides in *S. gordonii* biofilms, thereby altering total biofilm biomass and oral colonization of the murine oral cavity. Our data also show that SGO_2031 is widely prevalent and conserved among streptococci and appears to affect the acetylation of *S. gordonii* proteins.

## MATERIALS AND METHODS

### Bacterial strains, media, plasmids, and primers

Working stocks of bacterial strains were obtained from overnight cultures and stored in sterile glycerol at −80°C. *S. gordonii* DL1, wild-type (WT), and isogenic mutant derivatives were grown at 37°C in 5% CO_2_ using Brain Heart Infusion (BHI) broth or agar plates (Difco; Sparks, MD). Antibiotic supplementation was added as needed and is described throughout the manuscript.

### Construction of *S. gordonii* mutants

*S. gordonii* DL1 mutants were constructed as previously described using a markerless mutagenesis system (9). Gene deletion was confirmed by PCR. The primers used to delete SGO_2029, SGO_2030, and SGO_2031 are listed in Table 1.

**TABLE 1:** List of primers^*a*^

Locus	Primer name	Sequence	Source
SGO_2029			
	SGO_2029 UP F	GAC CAA CTT TGG AAG ATA AAG ACG C	This study
	SGO_2029 Up R - JHMD1	CTA TGC TAT GAG TGT TAT CGT TTC TCG TTT TCC ATT CTT GGG GTT TGG G	This study
	SGO_2029 Dn F - JHMD1	GTT ATC TAT TAT TTA ACG GGA GGA AAT AAG ATT AAC CAG AGC AAA GAT GCC C	This study
	SGO_2029 Dn R	CAA ATG AAA AGA GCA GCT AGA ACT GC	This study
	2029.qRT.fwd	CCTCTATGTCAGTGGCTGTATG	This study
	2029.qRT.rev	CTCCTCCAACTCTTGGGTATAAG	This study
SGO_2030			
	SGO_2030 UP F	GAA ACT AAA TAA GAA GCG GGA GTG	This study
	SGO_2030 Up R - JHMD1	CTA TGC TAT GAG TGT TAT CGT TTC TCG AGG CTT TCC TCA ATC TAT ATC AAT	This study
	SGO_2030 Dn F - JHMD1	GTT ATC TAT TAT TTA ACG GGA GGA AAT AAG GAT AGA GCT AGA TGG AGA	This study
	SGO_2030 Dn R	AAA ACG AAA GCA AAT ACG TC	This study
	2030.qRT.fwd	CACATGGAAGCTGGCTAAGA	This study
	2030.qRT.rev	TTCCTCGTCAACCGAGATAAAC	This study
SGO_2031			
	SGO_2031 UP F	ACC GAT TCT GCC GGT TGG	This study
	SGO_2031 Up R - JHMD1	CTA TGC TAT GAG TGT TAT CGT TTC TCG TCT ACC ATA CTT TAC TTC TTC TTG C	This study
	SGO_2031 Dn F - JHMD1	GTT ATC TAT TAT TTA ACG GGA GGA AAT AAT GAG G AA GCC TAT GAA GTT ACG	This study
	SGO_2031 Dn R	CCA AGT CTT TCA TAA TCT GCT CC	This study
SGO_2032			
	2032.qRT.fwd	AGACGATAAAGGTAAGGCTCAAG	This study
	2032.qRT.rev	GCTAGCCTTTGCCAGAAATTG	This study
JHMD1			
	JHMD1 F	CGA GAA ACG ATA ACA CTC ATA GCA TAG	(9)
	JHMD1 R	TTA TTT CCT CCC GTT AAA TAA TAG ATA AC	(9)
16S rRNA			
	16S.qRT.fwd	AAGCAACGCGAAGAACCTTA	(10)
	16S.qRT.rev	GTCTCGCTAGAGTGCCCAAC	(10)

^*a*^ The underlined sequence represents the JHMD1 portion of the primers.

### RNA isolation and RT-qPCR

Total RNA was isolated according to a modified protocol (9). In short, overnight cultures were diluted in 100-mm saliva-coated dishes (Sarstedt) for biofilm cells to an initial OD_600nm_= 0.1 in 10 mL of sterile BHI. Cultures were incubated for 16 to 18 h at 37°C in 5% CO_2_. Media (planktonic cultures) were collected from biofilm cultures and centrifuged at 4,300 rpm at 4°C for 5 min. Next, the supernatant was removed by aspiration, and cells were resuspended in 500 μL of TRIzol reagent (Ambion). The cells that remained attached to the surface (biofilm) were gently washed twice with 10 mL of cold phosphate-buffered saline (PBS) to remove loosely attached cells. After washing, 1 mL of TRIzol was added to coat the dish, and each biofilm was removed with a sterile cell scraper (Sarstedt). Next, samples were lysed as previously described (10). Following lysis, RNA was purified using the Direct-zol RNA MiniPrep Kit (Zymo Research) according to the manufacturer’s protocol. TURBO DNase (Life Technologies) was added to the purified total RNA following the manufacturer’s protocol to deplete genomic DNA. Total RNA was further repurified using the Quick-RNA MiniPrep kit (Zymo Research) and measured using a Denovix DS-11 FX +spectrophotometer. cDNA synthesis and real-time qPCR were performed as described (9), using 16S rRNA as the endogenous control gene.

### SGO_2031 complementation

The SGO_2031 deletion strain (Δ2031) was complemented by insertion of the endogenous SGO_2031 gene under the *ldh* promoter into the *ldh* locus of the *S. gordonii* genome. SGO_2031 was amplified from the WT *S. gordonii* strain using primer pair SGO_2031*-*Fwd (5′-ATG GAT GTA GAA TTA AGG AAA C-3′) and SGO_2031*-*Rev (5′-TCA ATC TAT ATC AAT CCA ATA ACG-3′). The entire *ldh* sequence was amplified using primer pair *ldh*-Fwd (5′-CGT ACG GCC GTC GAC GTC CGT AAA AAA ATG CCT CTT TTC AC-3′) and *ldh*-Rev (5′-CTA GTC CTA GGC CGG CGC CCT CAA TCT ATA TCA ATC CAA TAA CG-3′). The *ldh* fragment was fused to SGO_2031 and inserted by Gibson assembly (New England Biolabs, Ipswich, MA) into pFW5 (a suicide vector in *S. gordonii*), previously digested with enzymes BamHI-F and SmaI (New England Biolabs, Ipswich, MA). The resulting plasmid, named pJO1, was transformed into *S. gordonii* for genomic integration.

### Saliva collection and preparation

Stimulated whole saliva was collected and processed as described (23) using protocols that were reviewed and approved by the Institutional Review Boards from the University of Minnesota (STUDY00016289). The sterile saliva was used to coat surfaces for biofilm growth and assays, protein extraction, and RNA isolation. Briefly, pooled human saliva was centrifuged at 4,300 rpm for 10 min at 4°C, and supernatants were aliquoted into Eppendorf tubes and centrifuged at 15K rpm for 20 min at 4°C. Next, the aliquots were pooled, and 200 μL of the clarified saliva was added to each microtiter plate well. The plates were incubated for 1 h at room temperature on an orbital shaker, and excess saliva was aspirated from each well. Lastly, the saliva-coated plates were sterilized by UV irradiation for 15 min (Spectroline UV Crosslinker FB-UVXL-1000 [Westbury, NY]).

### Crystal violet retention assay

Biofilm formation was assessed by crystal violet retention as previously described (24). First, bacteria were grown in BHI broth overnight (16 to 18 h) at 37°C and 5% CO_2_ in saliva-coated, 96-well plates (Corning). After overnight growth, the plates were inverted to remove the media. Next, the biofilm was stained with 50 μL 0.1% crystal violet for 15 min at room temperature, washed four times with PBS, and air-dried on paper towels for 2 min. To measure biomass, 200 μL acidified ethanol (4% 1N HCl/EtOH) was added to each well and incubated for 2 min. Next, the wells were gently mixed, and then 125 μL of the acidified ethanol solution from each well was transferred to a new flat-bottom 96-well plate for absorbance reading at 570 nm (BioTek Synergy 2 reader).

### *In silico* search for SGO_2031 homologs and phylogenetic tree construction

The following *S. gordonii* GNAT sequences were identified by searching for the GNAT domain (PF00583) in this species in Uniprot: SGO_0709, UniProt: A8AW51; SGO_1472, UniProt: A8AY91; SGO_1868, UniProt: A8AZC4; SGO_2030, UniProt: A8AZR8; SGO_2031, and UniProt: A8AZR9. tBLASTn searches were performed on NCBI’s non-redundant database to identify related sequences in *S. gordonii* that may not have been previously annotated and the presence or absence of orthologs in other species. The top BLAST hits for each species were chosen, limited to 100 results per search, including and excluding *S. gordonii* as a target species. Top hits were aligned using MAFFT v7.490 (25, 26) in Geneious Prime 2023.1.2 using default parameters. Alignments were checked by the eye, and fragments less than 100 amino acids or obviously unaligned sequences were removed. To check that sequences in the alignment were phylogenetically within the GNAT family, pseudo-maximum-likelihood trees were generated using FastTree v2.1.11 (27, 28) with default parameters in Geneious. Highly divergent orphan sequences were removed from the alignment and rechecked with FastTree. Once the alignment was finalized, a maximum-likelihood phylogenetic tree was constructed with IQtree2 v2.0.3 (29) using the Minnesota Supercomputing Institute’s (MSI) High-Performance Computing system at the University of Minnesota. The best-fit model of protein evolution (LG + G4) was auto-chosen based on the Bayesian Information Criterion (BIC) test. Support for the tree was calculated with 1,000 approximate SH-likelihood ratio test (aSH-LRT) replicates and 1,000 UltraFast Bootstrap (UFbs) replicates. Branches are conservatively considered well supported if support values for *both* aSH-LRT and UFbs were above 80% and 95%, respectively.

### Growth curve assay

Static overnight cultures of *S. gordonii* WT and SGO_2031 deletion strains were grown as described above, standardized to an initial cell density of OD_600nm_ = 0.1, and incubated at 37°C in 5% CO_2_. Every 45 min, the cultures were gently agitated, and the optical density (λ= 600 nm) was recorded for 11 h as previously described (23).

### Surface attachment assay

Attachment to saliva-coated surfaces was assessed as previously described (23). Standing overnight cultures of *S. gordonii* WT and SGO_2031 deletion strains were grown as described, standardized to a cell density of OD_600nm_ = 1, and incubated in BHI in 12-well saliva-coated plates (Corning) for 45 min at 37°C in 5% CO_2_. After incubation, the growth media were aspirated, and each well was washed twice with 1 mL of sterile PBS. Adherent cells were removed using sterile cell scrapers, resuspended in 1 mL of sterile PBS, serially diluted, and cultured on BHI plates to determine colony-forming units (CFU).

### Sample preparation for Western blot

*S. gordonii* WT and the SGO_2031 acetyl transferase mutant were grown on saliva-coated 6-well plates under 5% CO_2_ at 37°C overnight. Planktonic and biofilm fractions were harvested separately. The samples were treated with mutanolysin for 2 h at 37°C in spheroplasting buffer to separate the cell wall from the rest of the cell (protoplast) (30). After incubation, protoplasts and the cell wall were separated by centrifugation at 12,000 × *g* for 20 min at 4°C. Protoplasts were resuspended in 10 mL of bacterial lysis buffer (BugBuster [EMD Millipore Corp., Billerica, MA]), lysed by sonication (75 W for 8 min). Insoluble materials were removed by centrifugation (12,000 × *g*, 20 min, 4°C). Protein concentrations were determined using Pierce BCA Protein Assay (Thermo Scientific, Rockford, IL), and 100 µg of total protein was loaded per lane. The proteins were then separated by 4%–20% SDS-PAGE and transferred to a PVDF membrane. To detect acetylated proteins, a rabbit anti-acetylated-lysine polyclonal antibody (Cell Signaling Technology) was used at a 1:200 dilution. Blots were incubated overnight at 4°C. IRDye 800CW goat anti-rabbit secondary antibody (Li-Cor, Lincoln, NE) was used to visualize immunoreactive proteins. Total protein transferred onto the PVDF membrane was quantified using REVERT Total Protein Stain (Li-Cor, Lincoln, NE) according to the manufacturer’s instructions.

### Exopolysaccharide extraction and quantification

Exopolysaccharide (EPS) fractions were extracted from WT and SGO_2031 deletion biofilms grown on saliva-coated plates using the sodium hydroxide extraction method as previously described (31). Total carbohydrates in the EPS fractions were quantified using the phenol-sulfuric acid method as described with minor modifications (32). In brief, 25 µL of phenol (80% in H₂O vol/vol) was added to 1 mL aliquots of the EPS fractions and vortexed. Five milliliters of concentrated sulfuric acid was added, and the preparations were incubated at room temperature for 20 min. Absorbance at 450 nm was measured using a BioTek Synergy 2 reader.

### Confocal microscopy

For confocal laser scanning microscopy (CLSM), ibidi µ-slide 8-well coverslips (Cat. No. 80826) were coated with sterile saliva and rocked at room temperature at 75 rpm for 1 h. After coating, slides were UV sterilized for 15 min. Bacterial strains were grown overnight in BHI, and then the optical density was adjusted to 0.5. These adjusted cultures were diluted 1:50 into fresh BHI +1% sucrose with 1 μM dextran (Dextran, Alexa Fluor 647; 10,000 MW, Anionic, Fixable, Invitrogen Cat. No. D22914) and grown in 5% CO_2_ for 48 h. After incubation, biofilms were washed with 0.89% saline solution and stained for 15 min in the dark with 5 μM Syto 9 Green (Invitrogen Cat. No. S34854) before being washed and wet-imaged. Excitation and emission for the dyes were detected as per the manufacturer’s instructions. Biofilm images and calculations were acquired using the Nikon Ti2 confocal microscope with the Nikon C2plus camera equipped with a Plan Apo λ 60× oil objective with sequential illumination and a z-step of 0.5 µm. Images are representative, and data are the average of results from 10 biological replicates, with 2–3 images per replicate. Quantification of total and individual component (Syto9 and Dextran) biovolumes, as well as initial calculations, was performed using NIS-Elements 5.0 Imaging Software, and subsequent calculations were performed in Microsoft Excel (Microsoft 365 MSO, Version 2512). For each replicate, layers with less than 1% biovolume were excluded before the average Binary Area Fraction (BAFA), Binary Area (BAA), Measured Area, Layers, Biovolume (BAA * Layers * 0.5 µm), Biofilm Thickness (Layers * 0.5 µm), Fraction of Biomass (BAA/ sum of component BAA), and Total Biovolume (Sum of Component Biovolumes) were calculated. Technical replicate values were then averaged for each biological replicate and graphed using GraphPad Prism (Version 8.0.1).

### Mouse oral colonization

All animal experiments were reviewed and approved by the Institutional Animal Care and Use Committee of the University of Minnesota (Protocol ID: 2203-39907A) and performed on 4-week-old male and female littermates. C57BL/6J WT mice were bred in-house from stocks originally purchased from The Jackson Laboratory (Bar Harbor, ME, USA). Mice were housed in microisolator cages with food and water supplied *ad libitum* in a specific pathogen-free animal facility in compliance with the Association for Assessment and Accreditation of Laboratory Animal Care at the University of Minnesota. Bacterial growth and oral inoculation of our murine model were performed as previously described (33) and are depicted in Fig. 6A. At the end of the experiment, 3 days post-inoculation, the bacteria associated with the mice’s molar region were collected using an ultra-fine microapplicator (BeeSure – BE502T) by brushing the microapplicator tip against the tooth surfaces. The tips of the microapplicators were collected and stored at −80°C for DNA isolation.

### DNA extraction and sequencing

Bacterial DNA was isolated using the DNeasy PowerSoil Kit (QIAGEN, Hilden, Germany) according to the manufacturer’s instructions. DNA concentration was determined using a Qubit Fluorometer (Thermo Fisher Scientific, Waltham, MA, USA). Total genomic DNA (20 μL) was submitted to the University of Minnesota Genomics Center (UMGC, Minneapolis, MN, USA) for Illumina MiSeq sequencing of the V3–V4 hypervariable regions of the 16S rRNA, using the S-D-Bact-0341-b-S-17/S-D-Bact-0785-a-A-21 primer set (34). Dual-index sequencing was carried out with a read length of 301 nucleotides (nt), as previously described (35). Raw data are deposited in the Sequence Read Archive under accession numbers SRP398125 and SRP398127.

### Bioinformatics

Sequence processing was performed using mothur software (version 1.41.1) (36), as previously described (37). Sequences were paired-end-joined, trimmed for quality, and aligned using the SILVA database (version 138.1) for clustering. A 2% pre-cluster was used to remove erroneous sequences (38). Chimeras were identified and removed using the UCHIME program (version 4.2.40) (39). Operational taxonomic units (OTUs) were identified at 99% similarity using furthest-neighbor clustering and classified with the Ribosomal Database Project release (version 18) (40). Different databases were used for alignment and classification due to processing considerations described previously (41). Alpha diversity, measured as Shannon (42) and Chao1 (43) indices, was calculated using mothur to determine within-sample diversity. Differences in beta diversity were calculated using Bray-Curtis similarities to determine between-sample diversity (44).

### Statistical analysis

Differences in alpha diversity were determined using ANOVA. Differences in the relative abundances of genera were determined using the Kruskal-Wallis test and the Steel-Dwass-Critchlow-Fligner procedure for pairwise comparisons. Analysis of similarity (ANOSIM) (43) was used to evaluate differences in community composition. Ordination was performed using principal coordinate analysis (PCoA) (45). The correlation of predominant genus abundances with axis positions was performed using Spearman correlations. ANOVA and Kruskal-Wallis tests were completed using XLSTAT software (version 2020.3.1; Addinsoft, Belmont, MA, USA). ANOSIM, PCoA, and correlation analyses were performed using mothur. For CLSM, analysis was performed in GraphPad Prism. Outliers were identified using the ROUT method (robust regression followed by outlier identification) with Q = 1% (46) and removed before analyzing strain differences using unpaired *t*-tests. All statistical analyses were performed at ɑ = 0.05, with Bonferroni corrections for pairwise comparisons.

## RESULTS

### *S. gordonii* differentially expresses genes encoding putative GNATs in biofilms

**Fig 1:**
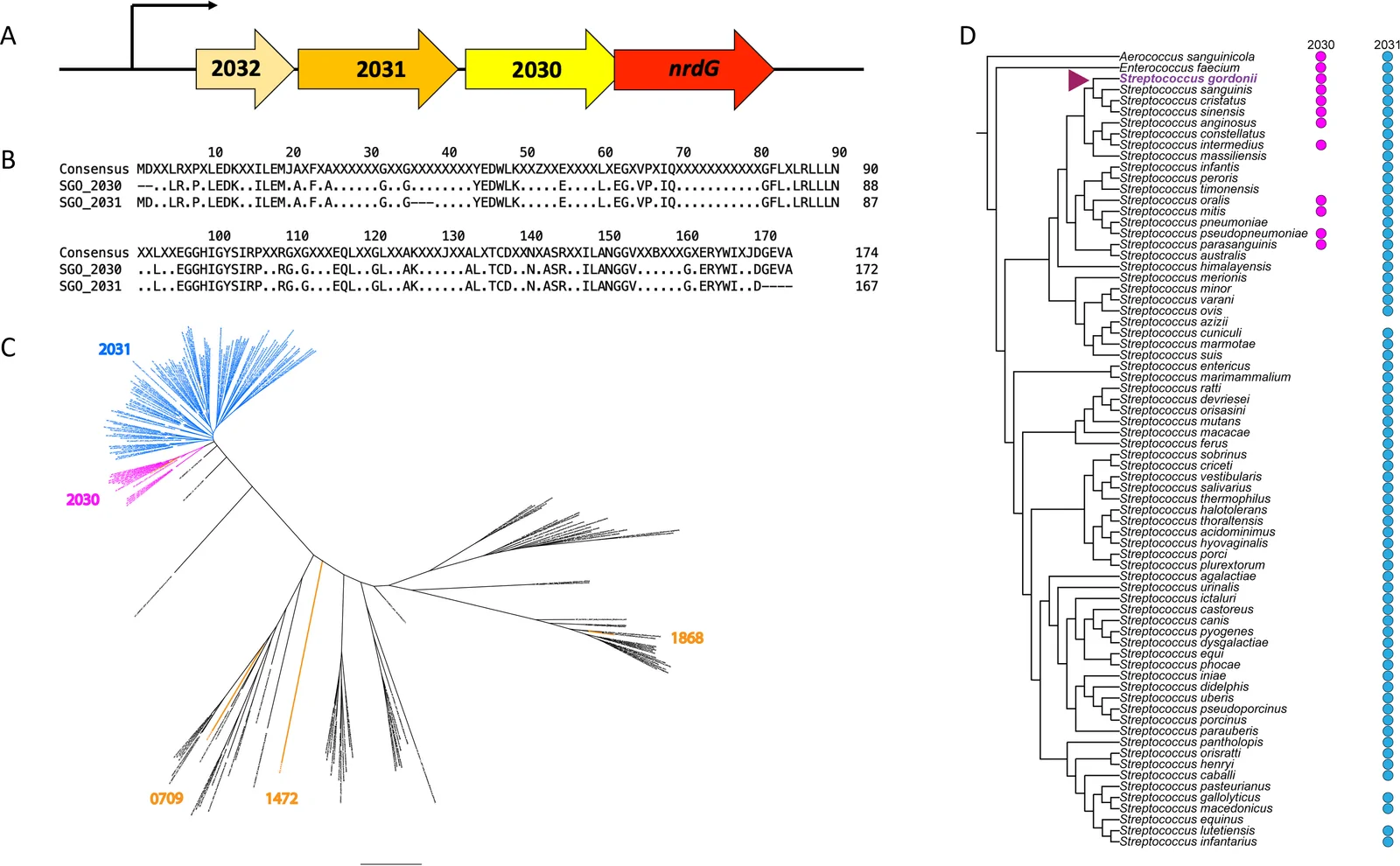
SGO_2030 and SGO_2031. (**A**) Schematic diagram of the genomic locus site containing the acetyltransferases SGO_2030 and SGO_2031. The bent arrow represents the putative promoter. (**B**) Amino acid sequence alignment of SGO_2030 and SGO_2031. (**C**) Unrooted, maximum-likelihood tree of related GNAT sequences. GNAT 2030 and 2031 are on a long branch distant from the closest related GNAT groups. *S. gordonii* sequences in outgroups are highlighted in orange. (**D**) Presence or absence of either SGO_2030 or SGO_2031 mapped onto the *Streptococcus* species tree with closely related outgroups.

We previously demonstrated that *S. gordonii* differentially expresses 63 genes when it forms biofilms on a polystyrene surface coated with the glycoprotein mucin MUC5B compared to a surface coated with MUC5B-depleted saliva (previously referred to as low-density protein fraction [LDP]) (11). Among the differentially expressed genes, two encode putative GCN5-like N-acetyltransferases (GNATs), SGO_2030 and SGO_2031. SGO_2030 and SGO_2031 are members of a putative four-gene operon that includes SGO_2032, which encodes a small (47 aa) conserved hypothetical protein, and SGO_2029, which shares homology to *Streptococcus pneumoniae nrdG,* encoding a ribonucleoside triphosphate reductase activator, required for capsular polysaccharide synthesis and export (47) (Fig. 1A).

At the amino acid level, SGO_2030 and SGO_2031 are 50% identical, with an overall 64% sequence similarity (Fig. 1B). Phylogenetic analysis of SGO_2030 and SGO_2031 shows that both proteins have highly divergent sequences compared to other *S. gordonii* GNATs, as indicated by the long branch length from the most closely related GNAT groups (orange). The branch leading to SGO_2030 and SGO_2031 is well supported; however, SGO_2030 and SGO_2031 are closely related to each other, and their distinct groups are not well supported (pink and blue groups, Fig. 1C), suggesting a recent duplication event. Both are found primarily in *Streptococci* and a few other related genera, unlike the broader representation of bacterial lineages seen in the next most closely related groups (orange, SGO_0709, SGO_1472, and SGO_1868). Orthologs of SGO_2031 are present in more *Streptococcus* species and other genera (*Aerococcus, Enterococcus, Salmonella, Peptoniphilus, Mycobacterium, Listeria, Shigella, Sphingomonas, Haemophilus, Aggregatibacter, Citrobacter, Rodentibacter*, and *Bacillus*) than those of SGO_2030 (*Aerococcus and Enterococcus*). Orthologs of SGO_2031 can also be found in several pathogenic organisms, including *Streptococcus pneumoniae, Streptococcus pyogenes*, and *Streptococcus mutans* (Fig. 1D).

### SGO_2031 contributes to biofilm formation

**Fig 2:**
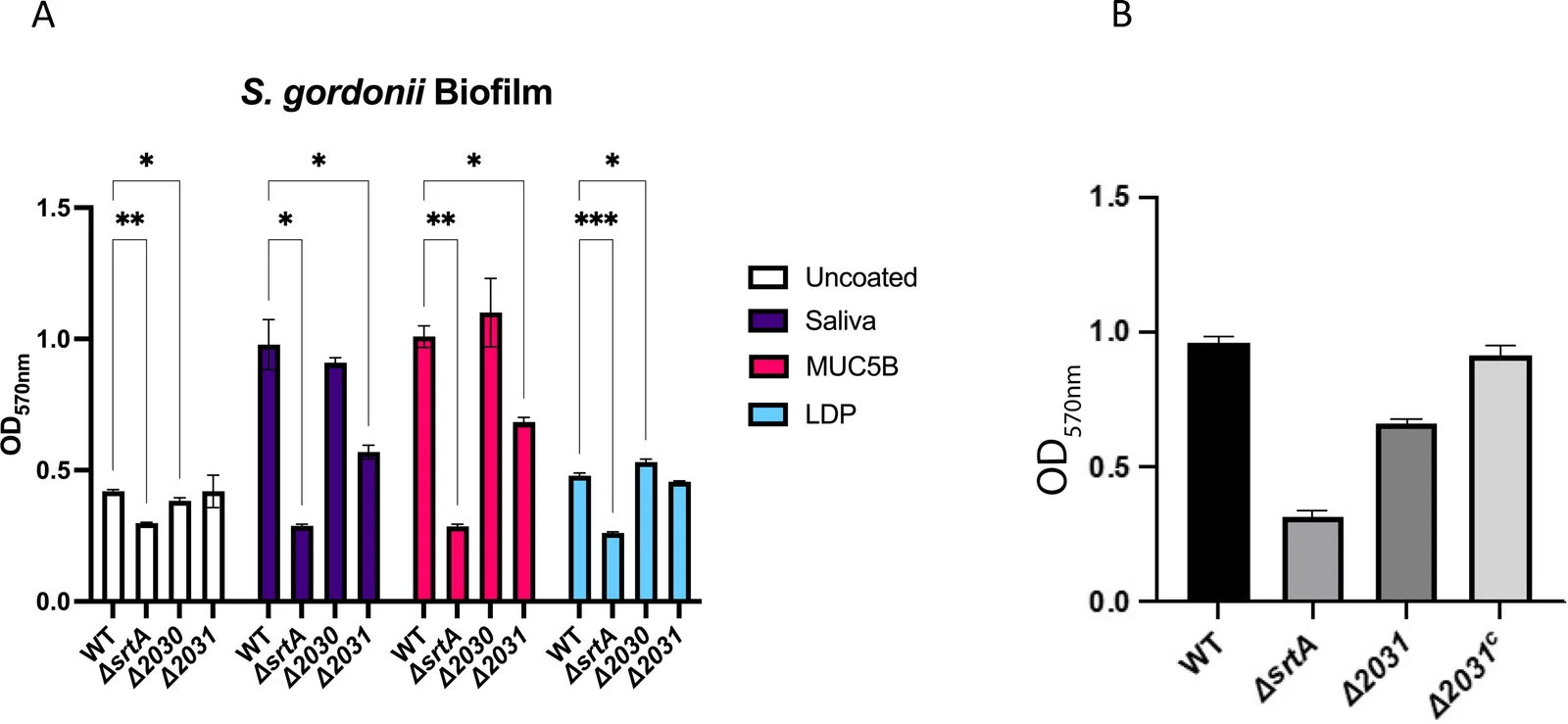
Quantification of *S. gordonii* biofilm formation on 96-well plates by crystal violet retention. (**A**) *S. gordonii* strain (WT, Δ*srtA*, Δ*2030, and* Δ*2031*) biofilms on 96-well plates uncoated or coated with saliva, MUC5B, or LDP. (**B**) *S. gordonii* strain (WT, Δ*srtA*, Δ*2031, and complemented* Δ*2031* (Δ*2031^c^*)) biofilms on 96-well plates coated with saliva. The data are reported as mean ± SD of OD_570nm_ readings (*n*  =  9; *P  <  0.05, **P  <  0.001, and ***P  <  0.001, ANOVA).

Given that SGO_2030 and SGO_2031 were differentially expressed under different biofilm growth conditions (i.e., MUC5B vs LDP) (11), we investigated whether these GNATs were involved in the formation of *S. gordonii* biofilms, using the WT *S. gordonii* as our positive control and our non-biofilm former strain, *∆srtA* (48, 49), as our negative control. Isogenic clean deletion mutants of SGO_2030 and SGO_2031 were allowed to form overnight biofilms on uncoated polystyrene surfaces or surfaces coated with sterile saliva, MUC5B, or MUC5B-depleted saliva (LDP). While no substantial defect was observed with the SGO_2030 deletion mutant, deletion of SGO_2031 led to an approximately 40% reduction in overnight biofilm biomass on both saliva- and MUC5B-coated surfaces (Fig. 2A), which could be restored to WT levels by complementation (SGO_2031^C^) and growth on saliva-coated surfaces (Fig. 2B). Consistent with the notion that surface binding is aided by *S. gordonii* interaction with the salivary glycoprotein MUC5B, we observed a significant decrease in biofilm biomass on uncoated and on LDP-coated surfaces (Fig. 2A). Thus, based on the conditions tested, SGO_2031, but not SGO_2030, seems to contribute to *S. gordonii* biofilm formation.

### SGO_2031 contributes to the biofilm matrix

**Fig 3:**
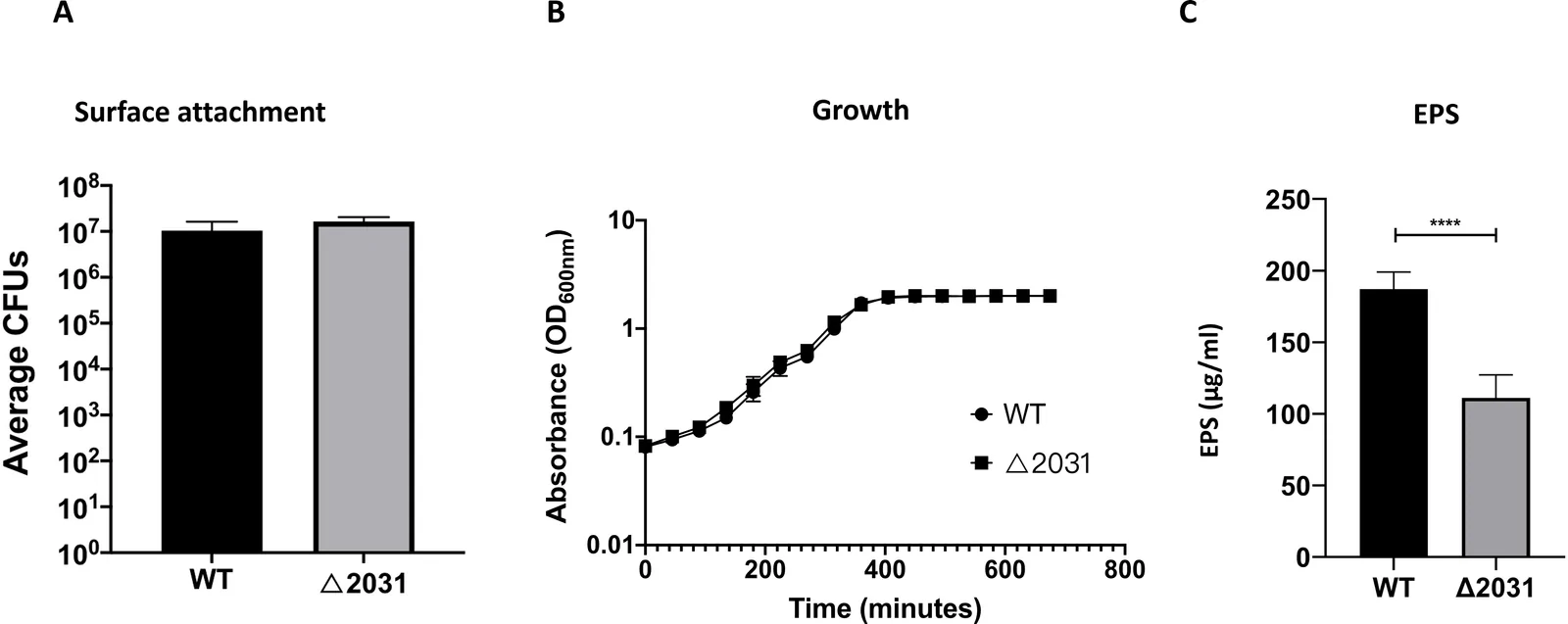
Characterization of SGO_2031 biofilm defect. (**A**) Quantification of the number of WT and Δ*2031* cells, represented as CFUs, that were able to attach to a saliva-coated polystyrene surface of a six-well plate (*n* = 6). (**B**) Growth curve of *S. gordonii* strains (WT and Δ2031) as determined by optical density (λ = 600  nm). Data represent means of results from three independent biological replicates (± standard deviations [SD]). (**C**) Quantification of EPS from 18-h biofilms of the WT and Δ*2031* strains. The data are reported as mean ± SD of measurement readings (*n*  =  9, ****P  <  0.0001 *t*-test).

**Fig 4:**
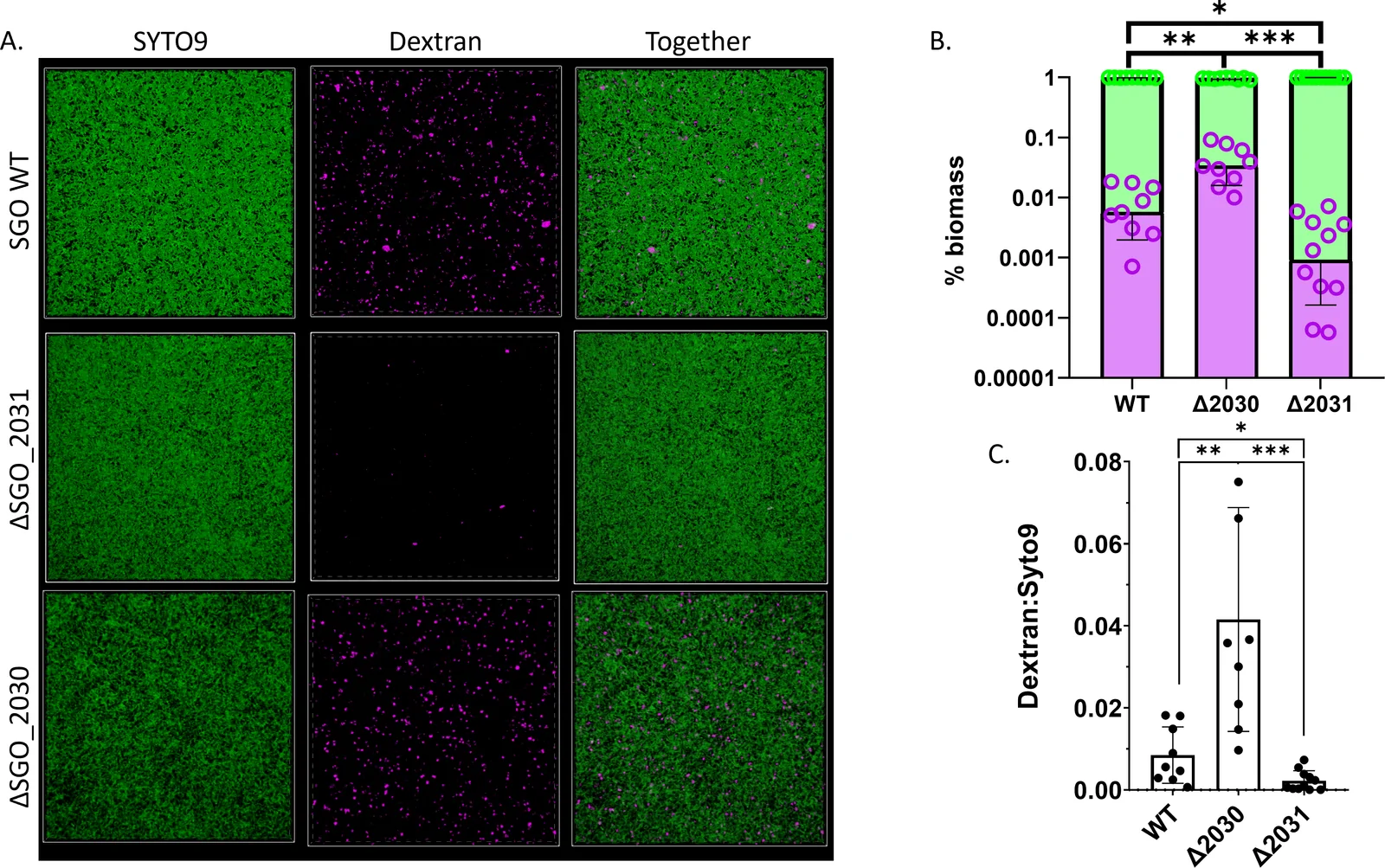
Confocal microscopy of *S. gordonii* biofilms. Biofilms of WT, Δ2031, and Δ2030 mutant strains were analyzed by CLSM. Biofilm cells were visualized using Syto 9 (green), and EPS was visualized by incorporation of a fluorescently labeled dextran (purple). (**A**) Representative images are shown. (**B**) Total and component biomass were quantified to calculate the component percentage of the biomass (**C**) and dextran:Syto9 ratio with individual values plotted as circles. Data from two to three technical replicates were averaged for each of 9–11 biological replicates. Two WT outliers were identified using the ROUT method with Q = 1% and removed before unpaired *t*-tests were used to compare strains (*n* = 9 for WT and Δ2030; *n* = 11 for Δ2031; *P  <  0.05, **P  <  0.005, and ***P  <  0.0005).

Biofilm formation is a multistep process that initiates with surface attachment, followed by the formation of microcolonies and the production of an extracellular matrix (50). Thus, to determine how SGO_2031 impacts biofilm formation, we investigated its effect on initial attachment to a saliva-coated surface, growth, and total exopolysaccharide (EPS) production. Compared to the WT, deletion of SGO_2031 did not affect initial attachment to saliva-coated surfaces (Fig. 3A) or growth in BHI broth over a period of 11 h (Fig. 3B), leading us to investigate if deletion of SGO_2031 affected the extracellular matrix, in particular, total EPS. Indeed, the loss of SGO_2031 resulted in a significant reduction in total carbohydrate concentration in the EPS fractions of the mutant biofilm compared to that of the wild-type (Fig. 3C). These results were confirmed by confocal laser scanning microscopy (Fig. 4; Fig. S1). While the total biovolume and thickness of biofilms were comparable between WT and SGO_2031 mutant (Fig. S1), the mutant showed significantly reduced EPS in its biofilm (Fig. 4). Interestingly, Δ2030 biofilms were significantly thicker than Δ2031 and had significantly higher dextran than both WT and Δ2031. Taken together, these experiments demonstrate that deletion of SGO_2031 does not seem to be involved in the initial surface attachment or growth rate of *S. gordonii* but rather impairs EPS production and/or export, causing the biofilm defect. Because SGO_2031 is located close to *nrdG* in the *S. gordonii* chromosome and since *nrdG* in *S. pneumoniae* has been shown to regulate capsular polysaccharide synthesis and export, we deleted the *S. gordonii nrdG* homolog and evaluated biofilm formation. However, no difference in biofilm biomass was observed when compared to the WT (Fig. S2A). To confirm that the phenotype seen in the SGO_2031 mutant was not due to the disruption of the expression of other genes in the operon, qRT-PCR was conducted, and it was confirmed that SGO_2029, SGO_2030, and SGO_2032 did not have a statistically different expression between the WT and the SGO_2031 deletion strains (Fig. S2B).

### SGO_2031 (SktA) protein acetylation profile

**Fig 5:**
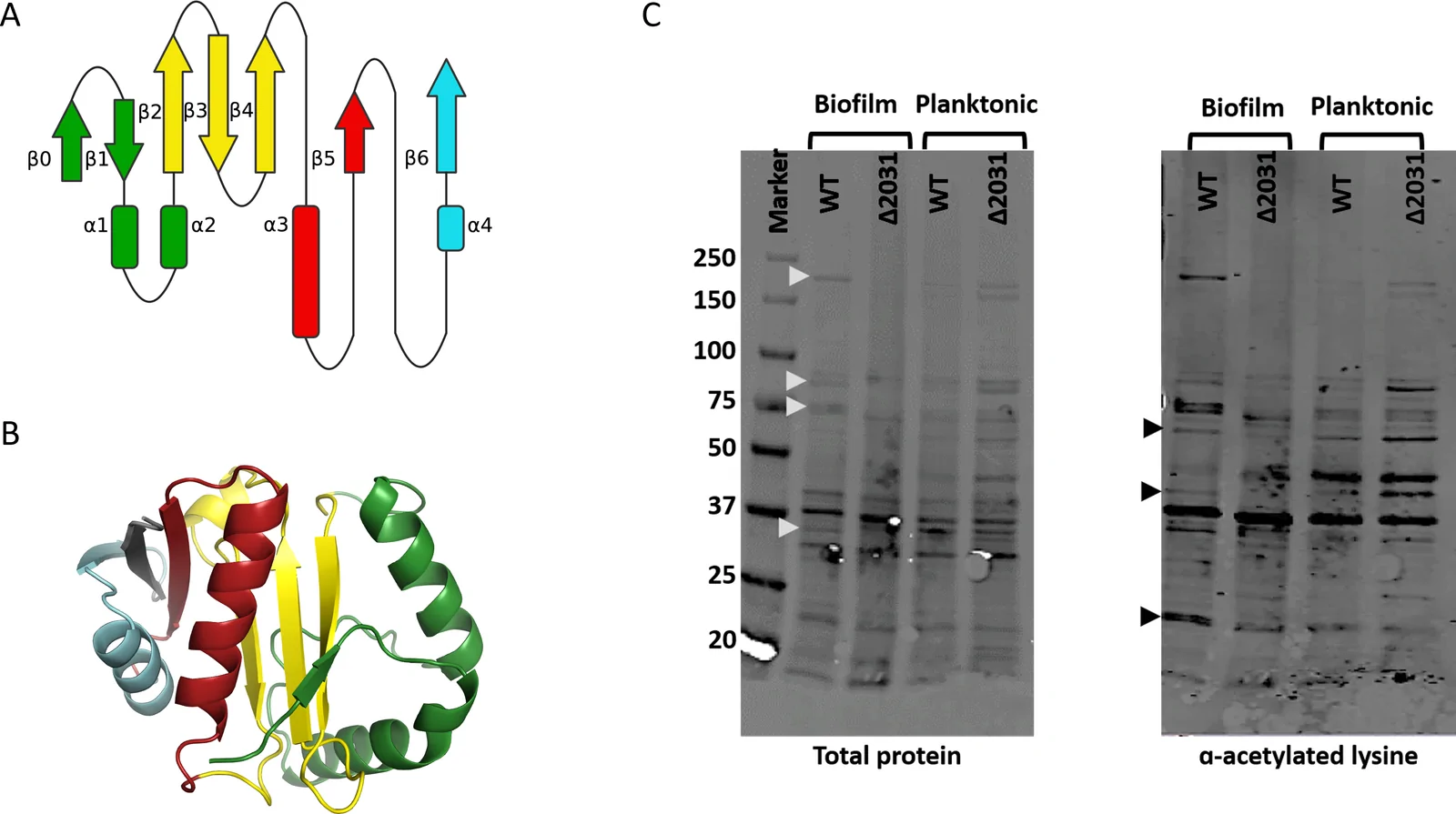
SGO_2031 is a GNAT. (**A**) Schematic diagram of the core secondary element of GNATs based on reference 51. (**B**) AlphaFold prediction of the structure of SGO_2031. The predicted secondary structures are colored to match panel **A**. (**C**) Soluble proteins (60 μg) from biofilm and planktonic cells of the WT and Δ2031 strains were resolved on a 4%–20%-gradient SDS-PAGE gel and transferred to a PVDF membrane. Total protein was visualized using REVERT Total Protein Stain (Li-Cor, Lincoln, NE), and acetylated proteins were visualized by Western immunoblot analysis. Arrows indicated differences in the signal intensity between biofilm samples of the WT and Δ2031 mutant strain.

Structurally, GNATs are relatively conserved, and members of this protein family display a core fold consisting of six or seven *β*-strands and four *α*-helices (51) (Fig. 5A). Structural prediction using AlphaFold suggests the presence of the conserved GNAT fold in SGO_2031 (Fig. 5B). To determine if SGO_2031 indeed functions as a bona fide protein acetyltransferase, affecting the overall protein acetylation profile of *S. gordonii*, we collected total protein from planktonic and biofilm fractions of cells of the wild-type and the SGO_2031 mutant. We assessed overall lysine acetylation by Western blot with an anti-acetyl lysine antibody. Consistent with its role as a protein acetyltransferase, deletion of SGO_2031 affected the band intensity and, therefore, the acetylation pattern of multiple proteins (Fig. 5C). Given the role of SGO_2031 as a lysine acetyltransferase and its prevalence and conservation among streptococci, from this point on, we will refer to SGO_2031 as streptococcal lysine (K) acetyltransferase A, or SktA.

### Loss of SktA decreases *S. gordonii* fitness in the murine oral cavity and alters the composition of the oral microbiome

**Fig 6:**
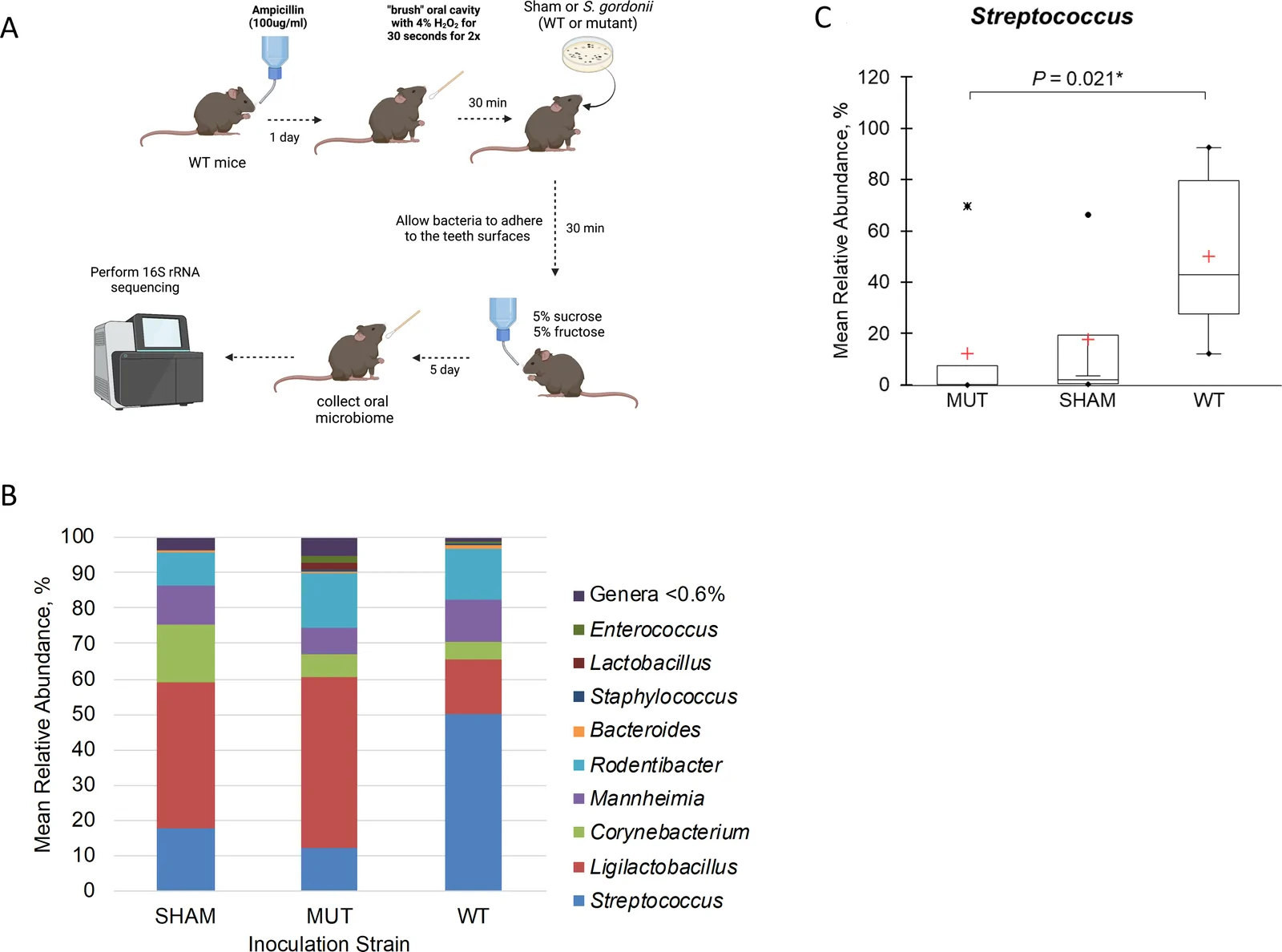
Oral colonization. (**A**) Diagram demonstrating the oral colonization protocol adapted from reference 33 and utilized in this study. (**B**) Relative abundances of bacterial genera detected in oral microbiota of mice inoculated with WT and *sktA* mutant strains or sham-inoculated. (**C**) Relative abundance of *Streptococcus* genus members detected in oral microbiota samples of mice inoculated with WT or *sktA* mutant strains or sham-inoculated (*n* = 8 for WT and *sktA* groups and *n* = 4 for the sham group).

The ability of *S. gordonii* to attach to surfaces and form biofilms is key to its survival in the oral cavity (52–54). Additionally, as an early colonizer of oral surfaces, *S. gordonii* is thought to play an important role in the biofilm composition (2, 55). To assess the role of SktA-dependent acetylation in oral colonization and its impact on oral microbiome composition, we orally inoculated WT C57BL/6 mice with equal numbers of WT and *sktA* mutant cells, following established protocols (33) and as illustrated in Fig. 6A.

Deletion of *sktA* led to an approximate 75% reduction in relative streptococcal abundance within the murine oral microbiome compared to mice inoculated with wild-type *S. gordonii* (from 50.1% to 12.1%) based on 16S rRNA gene sequencing of the V3–V4 regions. Notably, no significant relative differences were observed between the sham control and *sktA*-inoculated groups (Fig. 6B and C). We also observed that the relative abundance of *Ligilactobacillus* (recently reclassified from *Lactobacillus* [56]) in the murine oral cavity is inversely correlated with that of *Streptococcus* (Spearman’s *ρ* = −0.805, *P* < 0.0001; Fig. 6B; Fig. S3). Collectively, these data suggest that SktA, and therefore protein acetylation, enhances *S. gordonii* fitness within the complex multispecies environment of the murine oral cavity and that *S. gordonii* itself likely plays a key role in shaping the overall microbial community composition.

## DISCUSSION

*N*ε-lysine acetylation is a conserved posttranslational modification of proteins found across all domains of life, influencing diverse cellular processes (57). In bacteria, *N*ε-lysine acetylation has been demonstrated to occur on proteins involved in a wide range of physiological processes, including metabolism, transcription, DNA binding, and motility (58). In the present study, we propose a role for *N*ε-lysine acetylation, here referred to as protein acetylation, in *S. gordonii* biofilms. We show that the conserved GNAT SktA (formerly SGO_2031) likely influences *S. gordonii*’s production, secretion, or maintenance of extracellular polysaccharides, thereby affecting biofilm formation, oral colonization in a murine model, and overall community composition.

SktA is located within a four-gene locus that includes *nrdG* (SGO_2029), a ribonucleoside triphosphate reductase activator; *sktB* (SGO_2030), another putative GNAT; and a small 47-amino-acid conserved hypothetical protein (SGO_2032) (Fig. 1A). *nrdG* is the first gene of a conserved receptor polysaccharide gene cluster in *S. gordonii* and *S. sanguinis* (47). Homologs of this cluster have been implicated in the synthesis and export of capsular polysaccharides (CPS) in multiple streptococcal species (47). In *S. gordonii*, receptor polysaccharides can mediate cell-cell adhesion with other oral species (59), potentially contributing to dental plaque formation. The deletion of *nrdG* in *S. gordonii,* however*,* had no impact on single-species biofilm formation (Fig. S2A).

At the amino acid level, SktB and SktA are 50% identical and 64% similar to each other (Fig. 1B). Both are only found in streptococci and a few other related genera and are very different from the other putative GNATs found in the *S. gordonii* genome (Fig. 1C). This suggests an evolutionary pathway in which the ancestor of the GNAT SktA was itself the result of a duplication after the lineage split from other Firmicutes, and this ancestral locus duplicated again more recently in the oral Streptococcus lineage to give rise to SktB. The presence of SktB in *Aerococcus* and *Enterococcus*, but not in non-oral streptococci, could reflect horizontal gene transfer among these genera or a significant loss within *Streptococcus*.

Although similar in sequence, SktA and SktB may have distinct functions, at least when it comes to biofilm formation, since *sktA* deletion mutants showed about a 40% reduction in biofilm biomass on saliva- and MUC5B-coated surfaces, whereas *sktB* deletion had no noticeable effect on biofilm phenotype compared to WT. Additional experiments are ongoing to identify the acetylation targets and, therefore, potential physiological roles of these putative GNATs in *S. gordonii* and other streptococci.

Biofilms are complex microbial structures typically found on surfaces. A simplified view of the steps required for the development of these structures involves initial surface attachment, followed by cellular proliferation and the production of an extracellular polymeric matrix often composed of proteins, nucleic acids, and sugars (60). In *Streptococcus mutans*, acetylation of the glycosyl transferases GtfB and GtfC has been shown to affect EPS production and tooth decay in a rodent model (61). In this study, the loss of SktA does not appear to affect surface attachment, cellular proliferation, or biofilm biovolume and thickness in *S. gordonii*; however, a significant reduction in EPS was observed. Although the precise mechanism behind SktA's contribution to *S. gordonii* biofilms remains unclear, additional experiments are underway to identify the acetylation targets of SktA and its close homolog, but phenotypically distinct, SktB.

While *sktA* deletion only modestly impaired biofilm formation *in vitro*, this partial defect translated into a pronounced colonization failure in our *in vivo* oral colonization model. Specifically, no significant difference in the mean relative abundance of streptococci was observed between mice inoculated with the *sktA* mutant and sham-inoculated controls (Fig. 6C). As expected, the presence of *S. gordonii* in the murine oral cavity altered the overall microbial community structure (Fig. 6B; Fig. S3), with the relative abundance of *Ligilactobacillus* showing an inverse correlation with that of streptococci.

*Ligilactobacillus*, previously classified under *Lactobacillus* (56), is a group of lactic acid bacteria found in the oral cavity of many animals, including humans and mice (62). Species within this genus exhibit antagonistic interactions against streptococci. For example, *Ligilactobacillus salivarius* has been shown to inhibit single- and dual-species biofilms of *Streptococcus mutans* and *Candida albicans* (63). In contrast, the suppression of *Ligilactobacillus* by streptococci, as observed in our study, remains poorly characterized in the literature. Given that both genera compete for similar nutrient sources and adhesion sites in the oral niche (64), it is plausible that the relatively elevated inoculum of *S. gordonii* used in our colonization model shifted the ecological balance in its favor, thereby contributing to the observed relative reduction in *Ligilactobacillus* abundance.

### Concluding remarks

Our findings demonstrate that the acetyltransferase SktA is critical for *S. gordonii* biofilm formation and oral colonization. Loss of SktA seems to alter the acetylation profile of multiple cytoplasmic proteins in *S. gordonii*, impacting biofilm-relevant processes and disrupting colonization *in vivo*. This defect also seems to reshape the oral microbiota, notably reducing the relative abundance of *Ligilactobacillus*. These results highlight acetylation as a key regulatory mechanism in microbial fitness and interspecies dynamics.

## Data Availability

The authors confirm that the data supporting the findings of this study are available within the article and its supplemental materials. The microbiome data have been deposited in the Sequence Read Archive under accession numbers SRP398125 and SRP398127.

## SUPPLEMENTAL MATERIAL

Supplemental figures jb.00102-26-s0001.pdf Fig. S1 to S3.

ASM does not own the copyrights to Supplemental Material that may be linked to, or accessed through, an article. The authors have granted ASM a non-exclusive, world-wide license to publish the Supplemental Material files. Please contact the corresponding author directly for reuse.
