# Supplementary material for: Identification of a conserved GNAT-family lysine acetyltransferase in Streptococcus gordonii involved in biofilm formation and oral colonization
Source: J Bacteriol. 2026 Jul 27;208(8):e00102-26. doi: 10.1128/jb.00102-26 (PMC13492136; doi:10.1128/jb.00102-26)
Supplement: Supplementary file 1 [file jb.00102-26-s0001.pdf]

Supplemental information:

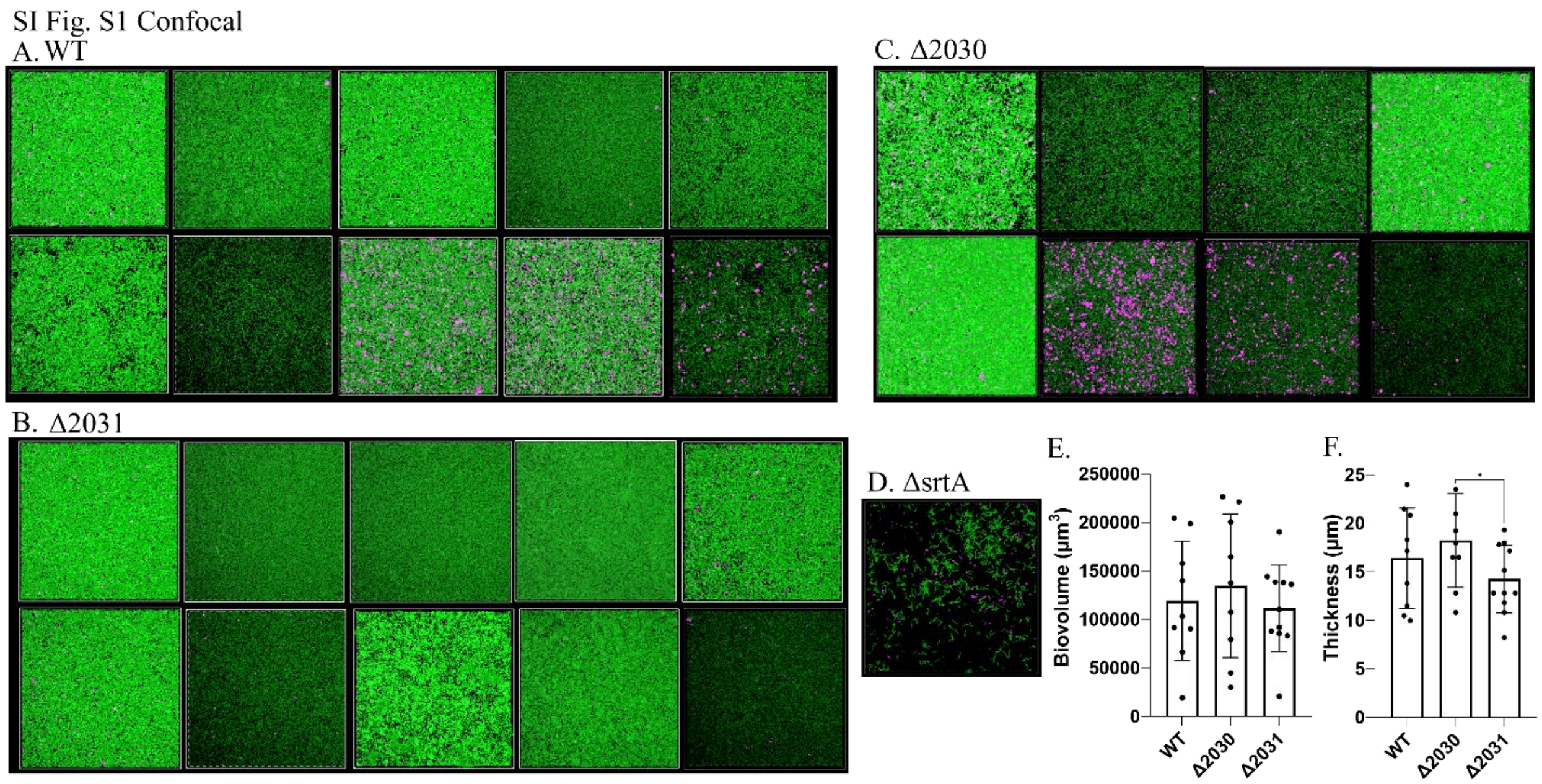

Supplemental Figure 1. Confocal microscopy of 48 h biofilms not shown in Figure 4. A combined image with Syto 9 (green) and Dextran (purple) is shown for each replicate of WT **A)**, ΔSGO_2031 **B**), ΔSGO_2030 **C**), or Δ*srtA* **D)**. Representative images were chosen from 2-3 technical replicates. Both biovolume **E)** and thickness **F)** of the biofilm were calculated and individual values plotted with significance determined by unpaired t-tests.

SI Fig. S2

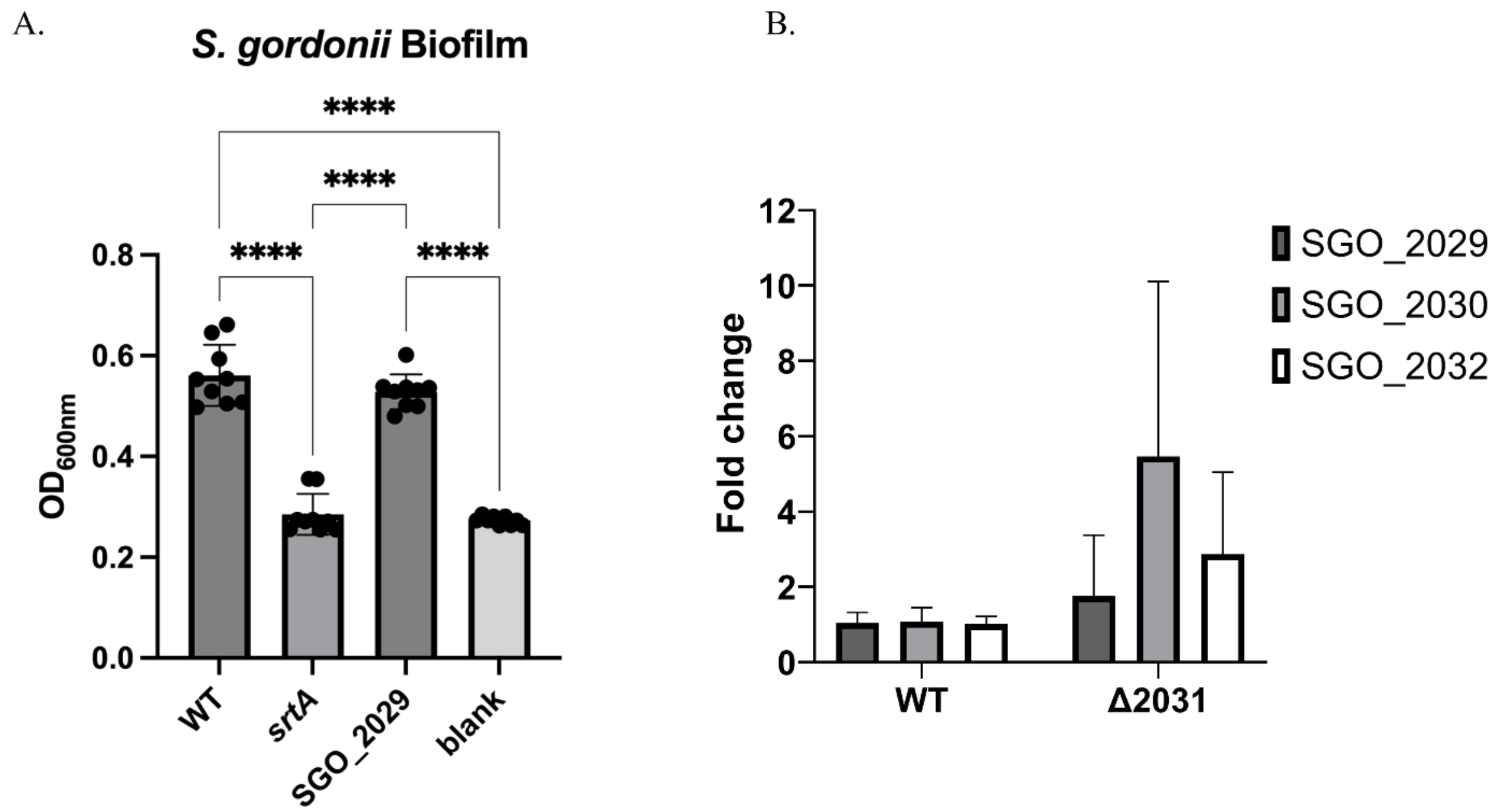

Supplemental Figure 2. Quantification of *S. gordonii* biofilm formation on 96-well plates by crystal violet retention **A)**. *S. gordonii* strains (WT, Δ*srtA* and Δ2029) biofilms on 96-well plates coated with saliva. Blank represents crystal violet retention values from well not inoculated with *S. gordonii*. The data are reported as mean ± SD of OD_{570nm} readings (*n* = 9; ****$P < 0.0001$, ANOVA). Expression of genes in the operon shown by qRT-PCR **B)**. RNA was extracted from planktonic cultures of WT and Δ2031 and the $\Delta\Delta C_T$ method (with C_T being the threshold cycle) determined expression fold changes compared to 16S as the control. The data are reported as mean ± SD (*n* = 5; t-tests).

SI Fig S3

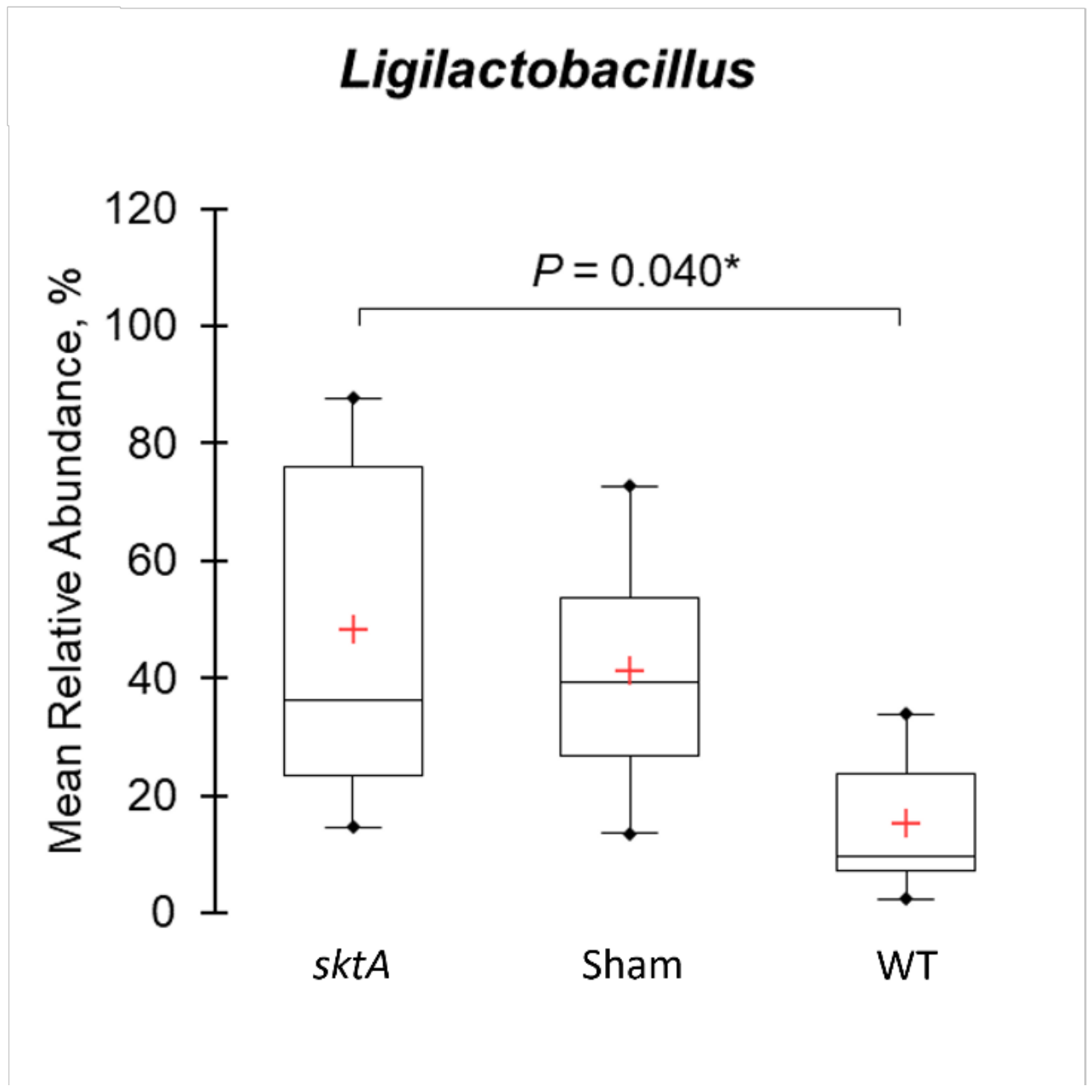

Supplemental Figure 3. Relative abundance of *Ligilactobacillus* genus members detected in oral microbiota samples of mice inoculated with WT or *sktA* mutant strains, or sham inoculated (*n* = 8 for WT and *sktA* groups, and *n* = 4 for sham group).
